# S‐allyl‐L‐cysteine protects hepatocytes from indomethacin‐induced apoptosis by attenuating endoplasmic reticulum stress

**DOI:** 10.1002/2211-5463.12945

**Published:** 2020-08-16

**Authors:** Peng Chen, Chen Chen, Mingdao Hu, Rui Cui, Feng Liu, Henghai Yu, Yuling Ren

**Affiliations:** ^1^ Department of Hepatopancreatobiliary Surgery The Second Affiliated Hospital of Kunming Medical University Kunming China; ^2^ Department of Ophthalmology The Second People's Hospital of Yunnan Province Kunming China

**Keywords:** apoptosis, endoplasmic reticulum stress, hepatocyte, indomethacin, S‐allyl‐l‐cysteine

## Abstract

Drug‐induced liver injury (DILI) can lead to acute liver failure, a lethal condition which may require liver transplantation. Hepatotoxicity associated with nonsteroidal anti‐inflammatory drugs (NSAIDs) accounts for ~ 10% of all DILI. In the current study, we determined whether indomethacin, one of the most commonly used NSAIDS, induced apoptosis in hepatocytes and investigated the underlying mechanism. Meanwhile, we investigated the protective effect of S‐allyl‐L‐cysteine (SAC), an active garlic derivative, on indomethacin‐induced hepatocyte apoptosis, and its implication on endoplasmic reticulum (ER) stress. We found that indomethacin triggered ER stress, as indicated by the elevated expression of phosphorylated eukaryotic translation initiation factor 2α (eIF2α), C/EBP homologous protein (CHOP) and spliced XBP1 in a rat liver BRL‐3A cell line. Following indomethacin treatment, caspase 3 activation and hepatocyte apoptosis were also observed. Inhibition of ER stress by chemical chaperone 4‐phenyl butyric acid alleviated cell apoptosis caused by indomethacin, indicating that ER stress is involved in indomethacin‐induced hepatocyte apoptosis. Moreover, SAC abated indomethacin‐induced eIF2α phosphorylation, inhibited CHOP upregulation and its nuclear translocation, abrogated the activation of caspase 3 and finally, protected hepatocytes from apoptosis. In conclusion, SAC protects indomethacin‐induced hepatocyte apoptosis through mitigating ER stress and may be suitable for development into a potential new therapeutic agent for the treatment of DILI.

## Graphical abstract

Hepatotoxicity associated with nonsteroidal anti‐inflammatory drugs (NSAIDs) accounts for ~ 10% of all drug‐induced liver injury (DILI). We found that indomethacin triggered endoplasmic reticulum (ER) stress and apoptosis in hepatocytes. S‐allyl‐l‐cysteine (SAC), an active garlic derivative, protects indomethacin‐induced hepatocyte apoptosis through mitigating ER stress. SAC may be suitable for development into a potential new therapeutic agent for the treatment of DILI.

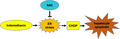

**Abbreviations**
4‐PBA: 4‐phenyl butyric acid
ALT: alanine aminotransferase
ATF4: activating transcription factor 4
CCK‐8: cell counting kit‐8
CHOP: C/EBP homologous protein
DAPI: 4′,6‐diamidino‐2‐phenylindole
DILI: drug‐induced liver injury
eIF2α: eukaryotic translation initiation factor 2α
ER: endoplasmic reticulum
IRE1α: inositol‐requiring enzyme 1α
LD50: median lethal dose
NSAIDs: nonsteroidal anti‐inflammatory drugs
PERK: PKR‐like ER kinase
ROS: reactive oxygen species
SAC: S‐allyl‐l‐cysteine
TUNEL: terminal deoxynucleotidyl transferase‐mediated uridine 5′‐triphosphate‐biotin nick end labeling
UPR: unfolded protein response
XBP1: X‐box binding protein 1

Drug‐induced liver injury (DILI) is a common cause of hepatitis and hospitalization worldwide [1]. Severe DILI leads to acute liver failure, a lethal condition which may require liver transplantation [2]. Indomethacin is among the most widely used nonsteroidal anti‐inflammatory drugs (NSAIDs) due to its antipyretic and analgesic properties. However, the hepatotoxicity associated with indomethacin is of concern to both physicians and patients. After chronic indomethacin intake, serum aminotransferase levels were found to be increased in 15% of patients [3]. Also, severe cases of acute hepatitis and death attributed to indomethacin therapy have been reported [3, 4, 5]. NSAIDS‐associated liver injury can be divide into three major types: hepatocellular, cholestatic and mixed, based on the ratio of plasma alanine aminotransferase to alkaline phosphatase (ALT/AP) [6]. Of these three types, indomethacin‐induced liver damage is mainly hepatocellular [7, 8]. The mechanisms how indomethacin impairs cell viability have been studied extensively, among which reactive oxygen species (ROS) generation, mitochondria dysfunction, and endoplasmic reticulum (ER) stress are essential [9, 10, 11].

Endoplasmic reticulum is an elaborate cellular network where protein folding and posttranslational modification take place. Perturbations of ER homeostasis lead to accumulation of unfolded or misfolded proteins, a situation termed ER stress [12]. To cope with ER stress, cells have evolved a signaling network known as the unfolded protein response (UPR). UPR either restores ER homeostasis or, if the restoration fails, triggers cell apoptosis [12, 13]. The C/EBP homologous protein (CHOP) is a principal mediator of ER stress‐associated cell apoptosis. Under ER stress, CHOP is induced predominantly via PKR‐like ER kinase (PERK)/eukaryotic translation initiation factor 2α (eIF2α)/activating transcription factor 4 (ATF4) signaling, and mediates apoptosis through mitochondrial pathway or death receptor pathway [14]. So far, CHOP has been shown to be implicated in NSAIDS‐associated cell apoptosis [11, 15, 16]. In human hepatoma cells, diclofenac and indomethacin induced ER stress and CHOP activation, resulting in cell death [15]. Additionally, in cervical cancer cells, overexpression of CHOP by plasmid transfection leaded to apoptosis without other stimuli, while knockdown of CHOP by siRNA alleviated apoptosis induced by celecoxib [16]. These studies provide powerful evidence for the implication of ER stress and CHOP activation in NSAIDS‐induced cell apoptosis.

S‐allyl‐l‐cysteine (SAC), an active and abundant garlic derivative, possesses antioxidant, antitumor and anti‐inflammatory characteristics [17, 18, 19, 20, 21]. Besides, compelling evidence demonstrates that SAC is hepatoprotective and neuroprotective [21, 22, 23, 24]. Our previous study revealed that SAC reduced ROS generation and reversed mitochondria dysfunction, thus protected hepatocytes from alcohol‐induced apoptosis [25]. Nevertheless, the impact of SAC on ER stress is still largely unknown. The current study aims to investigate whether SAC protects indomethacin‐induced hepatocyte apoptosis, and explore the implication of ER stress.

## Materials and methods

### Chemicals

Indomethacin (99.71%) was obtained from MedChemExpress LLC (Monmouth Junction, NJ, USA). SAC (≥ 98%) and 4‐phenyl butyric acid (4‐PBA; 99%) were obtained from Sigma‐Aldrich (St. Louis, MO, USA). Indomethacin was dissolved in DMSO, subpacked, and stored at −80 °C. SAC and 4‐PBA were dissolved in PBS, respectively, and aliquots were stored at −20 °C.

### Cell culture

BRL‐3A, a rat liver cell line, was obtained from Kunming Cell Bank of the Chinese Academy of Sciences (CAS; Kunming, China) and maintained at 37 °C with 5% CO_2_, as previously reported [25]. Cell passages 10–25 were used for all experiments.

### CCK‐8 assay

Cell viability was detected using CCK‐8 assay (Dojindo Laboratories, Kumamoto, Japan). Briefly, BRL‐3A cells were cultured in 96‐well plates and allowed 16 h to attach before being exposed to drugs. After treatment, medium was changed (100 µL per well) and 10 µL of CCK‐8 reagent was added to each well. Then, the plates were put back to cell incubator for 2 h, and absorbance was measured at 450 nm.

### Terminal deoxynucleotidyl transferase‐mediated uridine 5′‐triphosphate‐biotin nick end labeling assay

BRL‐3A cell apoptosis was detected by terminal deoxynucleotidyl transferase‐mediated uridine 5′‐triphosphate‐biotin nick end labeling (TUNEL) assay (In Situ Cell Death Detection Kit, TMR red; Roche Diagnostics, Indianapolis, IN, USA). Briefly, cells were seeded on coverslips in six‐well plates. After treatment, coverslips were washed and fixed with 4% paraformaldehyde, then permeabilized with 0.1% citrate buffer containing 0.1% Triton X‐100. After wash, coverslips were incubated with TUNEL reaction mix at 37 °C in dark for 1 h and rinsed with PBS for three times. Then, coverslips were mounted to a glass slide with a mounting medium contains 4′,6‐diamidino‐2‐phenylindole (DAPI) for nuclei staining. Slides were observed under a fluorescence microscope, and representative pictures were taken. TUNEL‐positive as well as total cell number were counted using imagej software (National Institute of Health, Bethesda, MD, USA) for quantification.

### Western blotting

Specific cellular protein levels were determined using western blot analysis. After treatment, BRL‐3A cells were lysed on ice with radioimmunoprecipitation assay buffer (Beijing ComWin Biotech, Beijing, China), and protein concentration was normalized using bicinchoninic acid kit (Beijing ComWin Biotech). Equal amounts of protein (20–40 µg) were separated by SDS/PAGE gel and electro‐transferred to nitrocellulose membranes. After blocking, membranes were probed overnight at 4 °C with following primary antibodies: anti‐X‐box binding protein 1 (XBP1s; 1 : 500), anti‐p‐eIF2α (1 : 1000), anti‐CHOP (1 : 1000), and anticleaved caspase 3 (1 : 500, Cell Signaling Technology, Danvers, MA, USA). After incubation with secondary antibodies, membranes were developed using chemiluminescence substrate (Beijing ComWin Biotech). The same membrane was stripped, washed, and incubated with anti‐β‐actin (1 : 5000; Proteintech, Rosemont, IL, USA) or anti‐eIF2α (1 : 1000; Cell Signaling Technology) for internal control. The protein bands were semiquantified by densitometry using imagej software (National Institute of Health).

### Reverse transcription PCR

Total RNA from BRL‐3A cells was extracted with the Eastep® Super Total RNA Extraction Kit (Promega, Madison, WI, USA) and cDNA was synthesized with the GoScript™ Reverse Transcription System (Promega) following the manufacturer's instructions. Reverse transcription PCR (RT‐PCR) was performed using the cDNA template and the GoTaq® Green Master Mix (Promega) as described previously [26]. The primers for rat *Xbp1* were as follows: 5′‐AGCAAGTGGTGGATTTGGAAGAAG‐3′ and 5′‐AGGGTCCAACTTGTCCAGAATG‐3′ [27]. PCR products were electrophoresed on a 2.5% agarose gel. The 346‐bp amplicon corresponds to the unspliced *Xbp1* mRNA, and the 320‐bp amplicon corresponds to the spliced *Xbp1* mRNA.

### Quantitative real‐time PCR

cDNA was synthesized as mentioned above. Quantitative real‐time PCR was carried out using the cDNA template and the GoTaq® qPCR Master Mix (Promega). The primers for rat spliced *Xbp1* were as follows: 5′‐GAGTCCGCAGCAGGTG‐3′ and 5′‐GCGTCAGAATCCATGGGA‐3′ [28]. The primers for rat *Chop* were as follows: 5′‐GAAAGCAGAAACCGGTCCAAT‐3′ and 5′‐GGATGAGATATAGGTGCCCCC‐3′ [27]. Data were analyzed by the comparative threshold cycle method, and glyceraldehyde phosphate dehydrogenase was used as the internal control.

### Immunocytochemistry

BRL‐3A cells were subcultured on coverslips in 6‐well plates. At the end of treatment, cells on coverslips were fixed with 4% PFA for 20 min, followed by permeabilization with 0.1% Triton X‐100 for 10 min. After blocking, coverslips were incubated with anticleaved caspase 3 antibody (1 : 100) or anti‐CHOP antibody (1 : 100; Cell Signaling Technology) at 4 °C overnight. Then, coverslips were washed and incubated with Alexa Fluor® 488 or 555‐conjugated secondary antibody (1 : 500; Cell Signaling Technology) at room temperature for 1 h in dark. After wash, coverslips were mounted to a slide (the mounting medium contains DAPI), and pictures were taken under a fluorescence microscope. Total and nuclear fluorescence was measured by imagej software (National Institute of Health).

### Statistical analysis

The quantitative data are presented as mean ± standard deviation (SD). graphpad prism 5.0 was used to analyze all data. Statistical analyses were carried out using analysis of variance (ANOVA). A *P*‐value of < 0.05 was considered to be statistically significant.

## Results

### Indomethacin induces apoptosis in hepatocytes

**Fig. 1:**
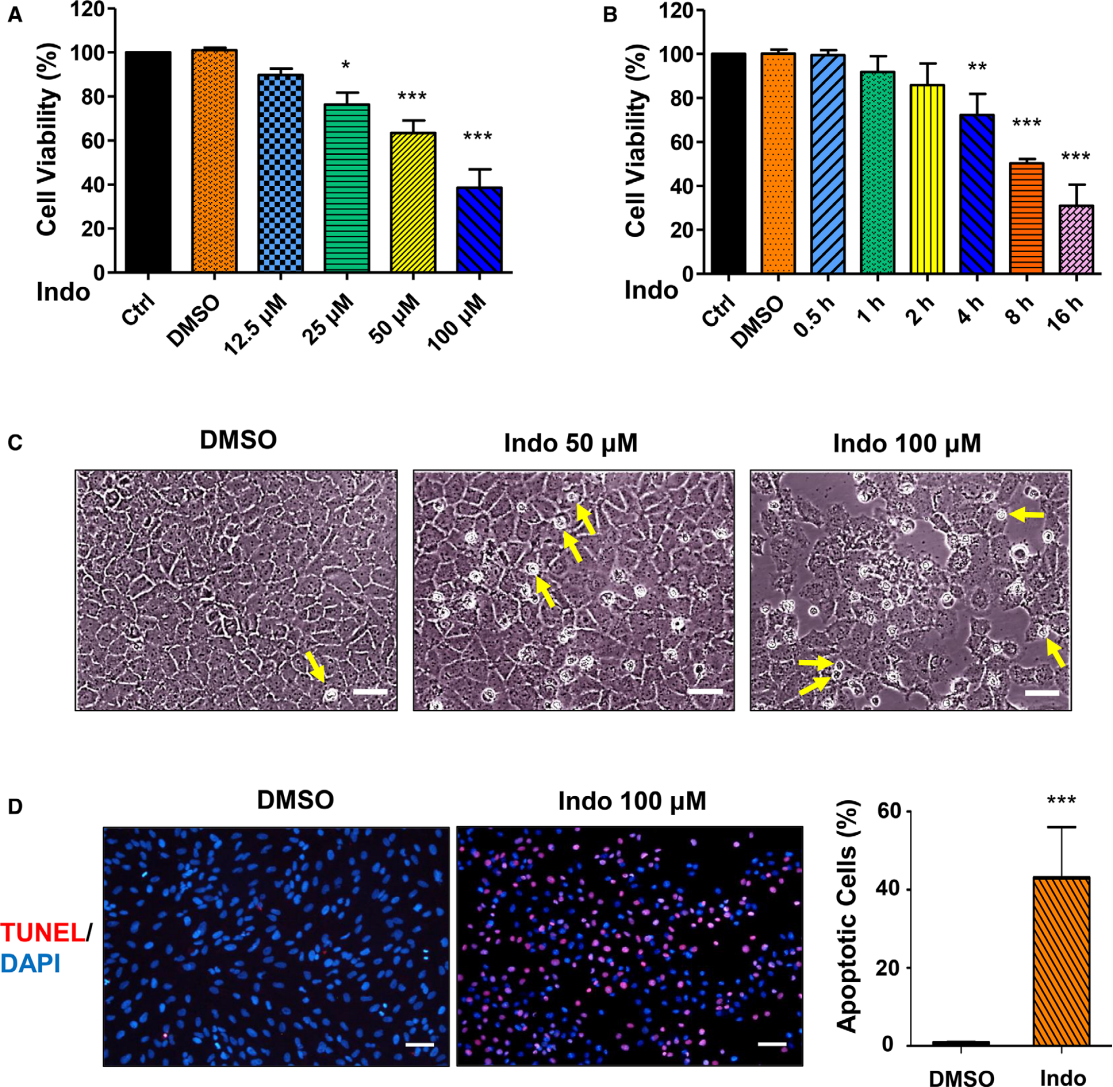
Indomethacin induces apoptosis in hepatocytes. (A) BRL‐3A cells were treated with different concentrations of indomethacin for 24 h. Cell viability was detected by CCK‐8 assay (*n* = 3, *P < 0.05, ***P < 0.001 vs. Ctrl). (B) BRL‐3A cells were exposed to 100 µm indomethacin for designated time. Cell viability was measured by CCK‐8 assay (*n* = 3, **P < 0.01, ***P < 0.001 vs. Ctrl). (C) BRL‐3A cells were challenged with 50 or 100 µm indomethacin for 24 h. Cell morphology was recorded under an invert phase contrast microscope (arrow: representatives of dead cells; bar: 50 µm). (D) Cells were treated with 100 µm indomethacin for 24 h. Apoptosis was detected by TUNEL assay (red: apoptotic cells; blue: cell nuclei stained with DAPI; bar: 50 µm) and quantified (*n* = 3 fields, ***P < 0.001 vs. Ctrl). Data were analyzed using ANOVA with Bonferroni's multiple comparison tests (A, B) or unpaired Student *t* test (D). Error bars indicate SD.

To demonstrate whether indomethacin induces hepatocyte apoptosis, we insulted BRL‐3A cells with different concentrations of indomethacin for 24 h. CCK‐8 assay revealed that indomethacin dose‐dependently impaired cell viability (Fig. 1A). Next, we exposed cells to 100 μm indomethacin for designated time. We found that cell viability decreased significantly since 4‐h exposure, and deteriorated as exposure time prolonged (Fig. 1B). Then, we recorded cell morphology under an invert phase contrast microscope after 24‐h indomethacin treatment. The pictures revealed that indomethacin increased dead cell (small round cell) number, and decreased total cell number (Fig. 1C). Furthermore, we detected BRL‐3A cell apoptosis by TUNEL staining. TUNEL assay stains genomic DNA fragmentation in apoptotic cells and cellular DNA damage in injured cells [29]. We found that indomethacin remarkably increased TUNEL‐positive (red) cell number (Fig. 1D). All these data demonstrate that indomethacin induces apoptosis in hepatocytes.

### Indomethacin induces ER stress in hepatocytes

**Fig. 2:**
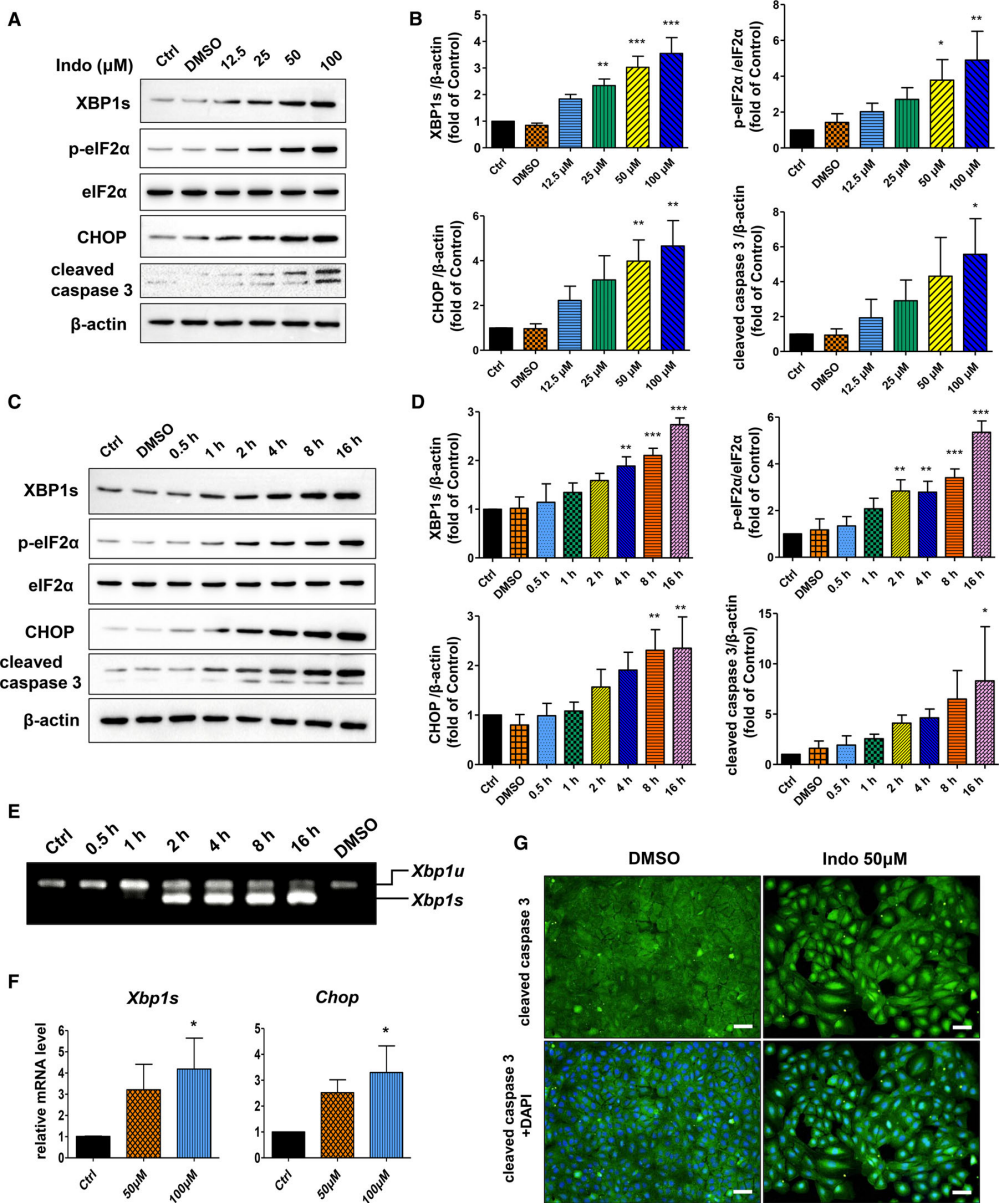
Indomethacin induces ER stress in hepatocytes. BRL‐3A cells were exposed to various concentrations of indomethacin for 8 h. Protein levels of spliced XBP1, phosphorylated eIF2α, total eIF2α, CHOP, and cleaved caspase 3 were determined by western blot (A) and analyzed by densitometry (B) (*n* = 3, *P < 0.05, **P < 0.01, ***P < 0.001 vs. Ctrl). BRL‐3A cells were treated with 50 µm indomethacin for designated time, and expressions of the same proteins were detected by western blot (C) and analyzed by densitometry (D) (*n* = 3, *P < 0.05, **P < 0.01, ***P < 0.001 vs. Ctrl). (E) BRL‐3A cells were exposed to 50 µm indomethacin for designated time, *Xbp1* mRNA splicing was detected by RT‐PCR (*n* = 3, *Xbp1u*, unspliced *Xbp1*; *Xbp1s*, spliced *Xbp1*). (F) Cells were treated with 50 or 100 µm indomethacin for 4 h, and mRNA levels of spliced *Xbp1* and *Chop* were determined by quantitative real‐time PCR (*n* = 3, *P < 0.05 vs. Ctrl). (G) BRL‐3A cells were exposed to 50 µm indomethacin for 16 h. Expression and distribution of cleaved caspase 3 was detected by immunocytochemistry (green: cleaved caspase 3; blue: cell nuclei stained with DAPI; bar: 50 µm). Data were analyzed using ANOVA with Bonferroni's multiple comparison tests. Error bars indicate SD.

To determine whether indomethacin triggers ER stress in hepatocytes, we exposed BRL‐3A cells to different concentrations of indomethacin for 8 h. The protein levels of several ER‐associated factors (spliced XBP1, phosphorylated eIF2α, CHOP) as well as cleaved caspase 3 were detected by western blot analysis. We found the expressions of these proteins increased dose‐dependently (Fig. 2A,B). Next, we treated BRL‐3A cells with 50 μm indomethacin for designated time. As expected, expressions of spliced XBP1, phosphorylated eIF2α, CHOP, and cleaved caspase 3 all increased time‐dependently (Fig. 2C,D). Moreover, *Xbp1* mRNA splicing was induced as early as 2 h after indomethacin exposure, as demonstrated by RT‐PCR (Fig. 2E). In parallel, the mRNA levels of spliced *Xbp1* and *Chop* both increased significantly after indomethacin treatment (Fig. 2F). To further explore the expression and distribution of cleaved caspase 3, we employed immunocytochemistry. We found that cleaved caspase 3 was remarkably induced in the nucleus after indomethacin treatment (Fig. 2G). Our data suggest that indomethacin triggers ER stress and apoptosis in hepatocytes.

### Inhibition of ER stress attenuates indomethacin‐induced hepatocyte apoptosis

**Fig. 3:**
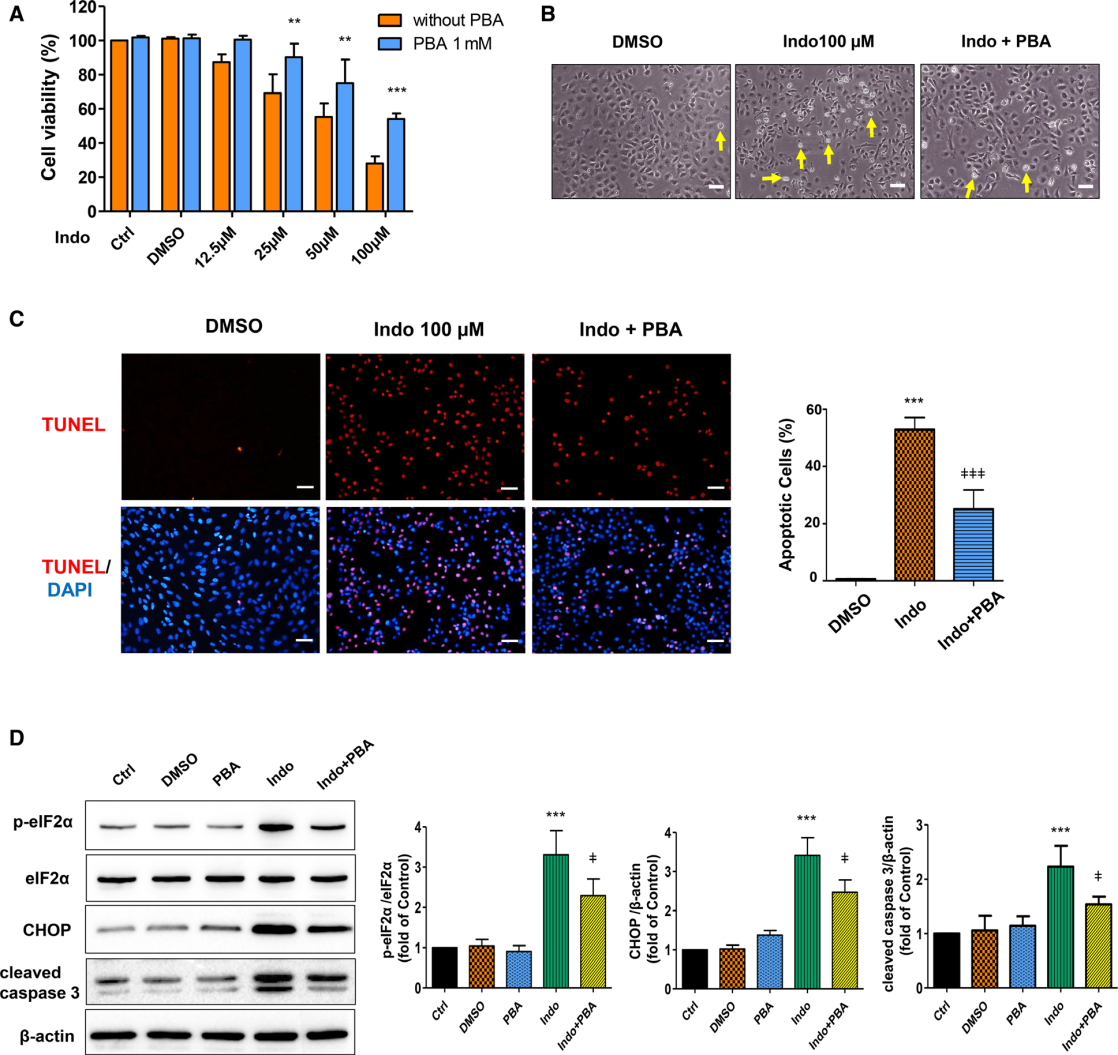
Inhibition of ER stress alleviates indomethacin‐induced hepatocyte apoptosis. BRL‐3A cells were pretreated with 1mM 4‐PBA for 24 h, followed by exposing to indomethacin for another 24 h. (A) Cell viability was detected by CCK‐8 assay (*n* = 3, **P < 0.01, ***P < 0.001 vs. cells treated with indomethacin only). (B) Cell morphology was recorded under an invert phase contrast microscope (*n* = 3, arrow: representatives of dead cells; bar: 50 µm). (C) BRL‐3A cell apoptosis was detected by TUNEL assay (red: apoptotic cells; blue: cell nuclei stained with DAPI; bar: 50 µm) and quantified (*n* = 3 fields, ***P < 0.001 vs. Ctrl, ^ǂǂǂ^ *P* < 0.001 vs. cells treated with indomethacin only). (D) BRL‐3A cells were pretreated with 1mM 4‐PBA for 24 h, then exposed to 50 µm indomethacin for 16 h. Protein levels of phosphorylated eIF2α, total eIF2α, CHOP, and cleaved caspase 3 were detected by western blot and analyzed by densitometry (*n* = 3, ***P < 0.001 vs. Ctrl, ^ǂ^ *P* < 0.05 vs. cells treated with indomethacin only). Data were analyzed using ANOVA. Error bars indicate SD.

4‐PBA is a chemical chaperone that helps protein folding, prevents protein aggregation in the ER, thus alleviates ER stress in various cells [30, 31, 32]. To find out whether ER stress is required for indomethacin‐induced hepatocyte apoptosis, we pretreated BRL‐3A cells with 4‐PBA before exposing the cells to different concentrations of indomethacin. CCK‐8 assay demonstrated a significant improvement of cell viability upon 4‐PBA pretreatment in indomethacin‐insulted BRL‐3A cells (Fig. 3A). In the mean time, cell morphology change induced by indomethacin was partially reversed by 4‐PBA pretreatment (Fig. 3B). Moreover, TUNEL assay confirmed the protective role of 4‐PBA as indomethacin‐induced cell apoptosis was alleviated upon 4‐PBA pretreatment (Fig. 3C). As CHOP is widely considered to be a principal mediator of ER stress‐associated apoptosis, we detected the expressions of CHOP as well as phosphorylated eIF2α and active caspase 3 (cleaved caspase 3) by western blot analysis. In line with other results, we found that eIF2α phosphorylation, CHOP upregulation and caspase 3 activation induced by indomethacin were all mitigated by 4‐PBA pretreatment (Fig. 3D). These findings indicate that ER stress is essential in indomethacin‐induced hepatocyte apoptosis.

### SAC rescues hepatocytes from indomethacin‐induced apoptosis

**Fig. 4:**
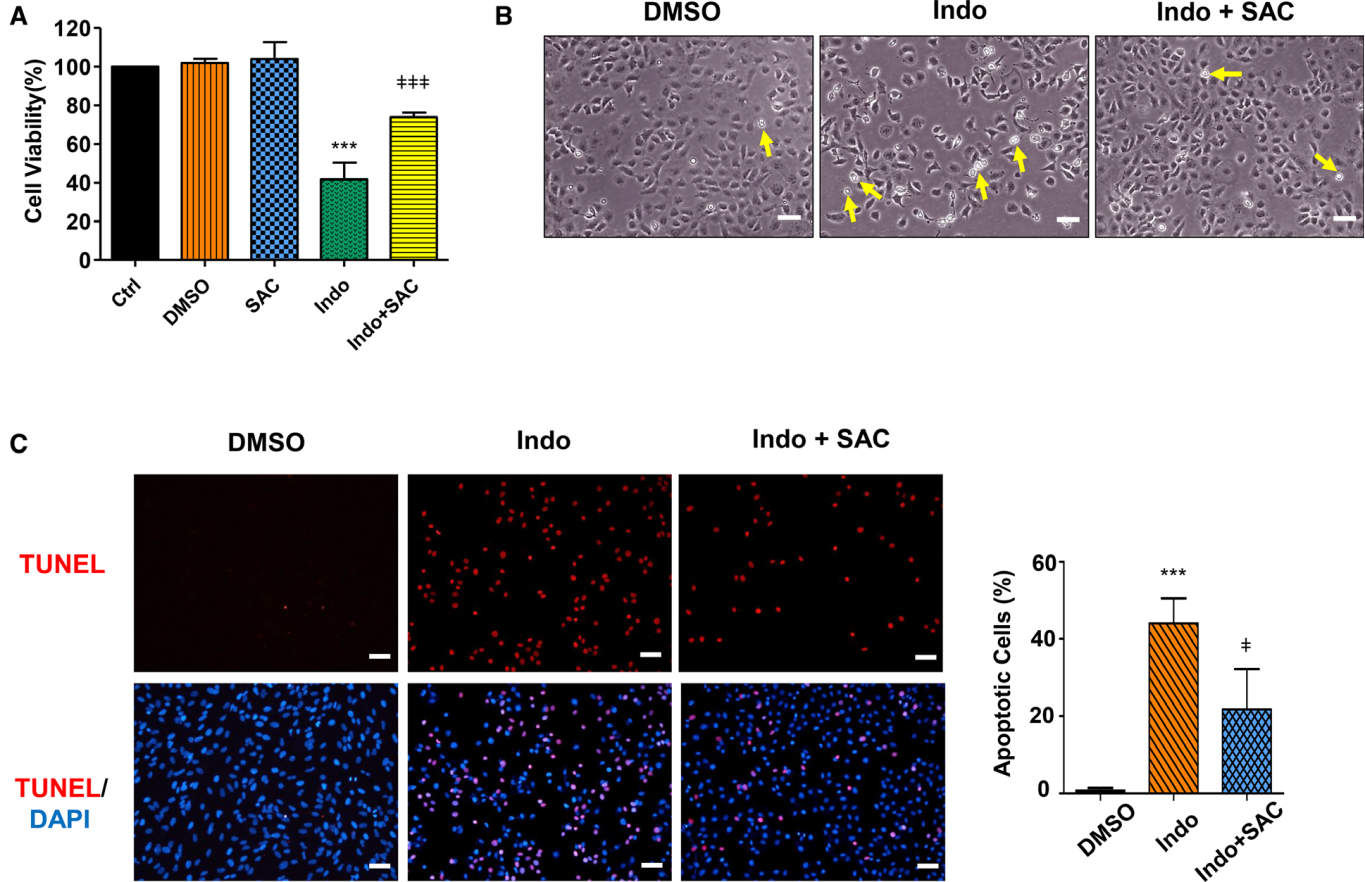
SAC rescues BRL‐3A cells from indomethacin‐induced apoptosis. BRL‐3A cells were pretreated with 50 µm SAC overnight (16 h), followed by exposing to 100 µm indomethacin for 24 h. (A) Cell viability was detected by CCK‐8 assay (*n* = 3, ***P < 0.001 vs. Ctrl, ^ǂǂǂ^ *P* < 0.001 vs. cells treated with indomethacin only). (B) Cell morphology was recorded under an invert phase contrast microscope (*n* = 3, arrow: representatives of dead cells; bar: 50 µm). (C) Apoptosis was detected by TUNEL assay (red: apoptotic cells; blue: cell nuclei stained with DAPI; bar: 50 µm) and quantified (*n* = 3 fields, ***P < 0.001 vs. Ctrl, ^ǂ^ *P* < 0.05 vs. cells treated with indomethacin only). Data were analyzed using ANOVA with Bonferroni's multiple comparison tests. Error bars indicate SD.

To demonstrate whether SAC alleviates indomethacin‐induced hepatocyte apoptosis, we pretreated BRL‐3A cells with SAC before exposing cells to indomethacin. CCK‐8 assay revealed that cell viability was decreased to 41.7% of the Control after cells were insulted by indomethacin for 24 h. Nevertheless, pretreatment of SAC restored cell viability to 73.9% of the Control (Fig. 4A). In parallel, SAC mitigated indomethacin‐induced cell morphology change and cell number decrease (Fig. 4B). Furthermore, SAC remarkably alleviated cellular DNA fragmentation and DNA damage in indomethacin‐treated cells, as demonstrated by the reduced number of TUNEL‐positive cells (Fig. 4C). All these results demonstrate that SAC protects hepatocytes from indomethacin‐induced apoptosis.

### SAC protects hepatocyte apoptosis through mitigating ER stress

**Fig. 5:**
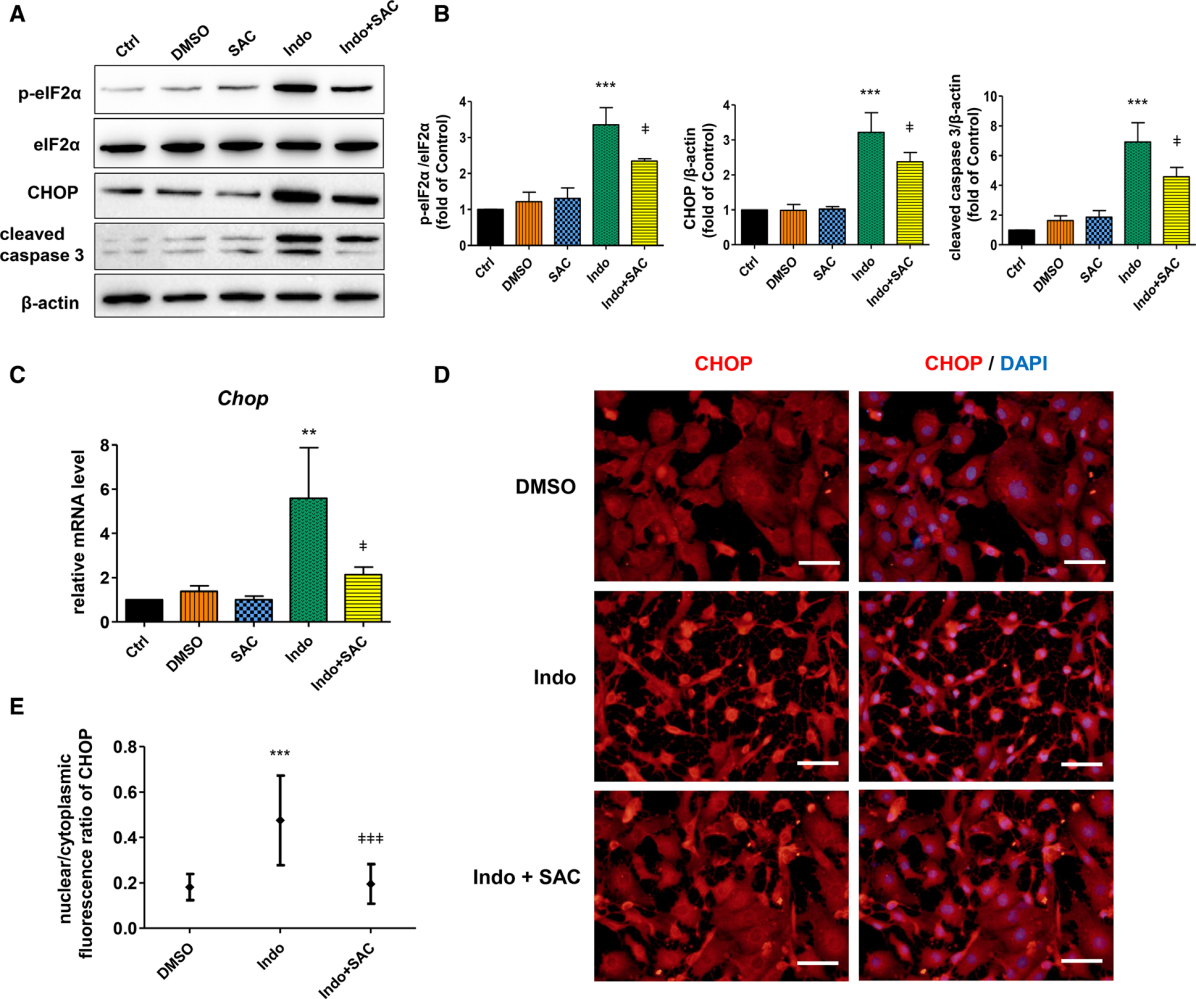
SAC protects hepatocyte apoptosis through mitigating ER stress. BRL‐3A cells were pretreated with 50 µm SAC overnight (16 h), followed by exposing to 50 µm indomethacin for another 16 h. Protein levels of phosphorylated eIF2α, total eIF2α, CHOP, and cleaved caspase 3 were determined by western blot (A) and analyzed by densitometry (B) (*n* = 3, ***P < 0.001 vs. Ctrl, ^ǂ^ *P* < 0.05 vs. cells treated with indomethacin only). (C) BRL‐3A cells were pretreated with 50 µm SAC overnight (16 h), then exposed to 100 µm indomethacin for 4 h. *Chop* mRNA level was determined by quantitative real‐time PCR (*n* = 3, **P < 0.01 vs. Ctrl, ^ǂ^ *P* < 0.05 vs. cells treated with indomethacin only). (D) Cells were pretreated with 50 µm SAC overnight (16 h), then exposed to 100 µm indomethacin for 24 h. Expression and distribution of CHOP was detected by immunocytochemistry (*n* = 3, red: CHOP; blue: cell nuclei stained with DAPI; bar: 50 µm). Total and nuclear CHOP fluorescence was measured by imagej software, and the nuclear/cytoplasmic ratio was calculated (E) (*n* = 20 cells, ***P < 0.001 vs. Ctrl, ^ǂǂǂ^ *P* < 0.001 vs. cells treated with indomethacin only). Data were analyzed using ANOVA with Bonferroni's multiple comparison tests. Error bars indicate SD.

**Fig. 6:**
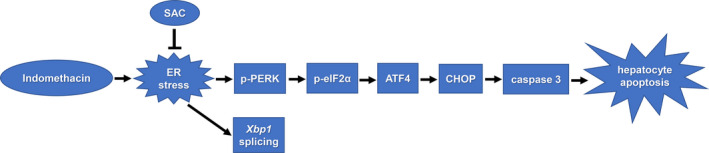
Schematic of the mechanism how SAC protects hepatocytes from indomethacin‐induced apoptosis. Indomethacin evokes ER stress, increases CHOP expression via PERK/eIF2α/ATF4 pathway, and induces caspase 3 activation resulting in hepatocyte apoptosis. SAC suppresses ER stress and rescues hepatocytes from apoptosis.

To further elucidate the mechanism by which SAC protects hepatocyte apoptosis, we explored the influence of SAC on the expressions of phosphorylated eIF2α, CHOP, and cleaved caspase 3 in indomethacin‐insulted BRL‐3A cells. We found that SAC *per se* had no effects on the expressions of these proteins under normal condition. However, upon SAC pretreatment, the induction of phosphorylated eIF2α, CHOP, and cleaved caspase 3 by indomethacin was mitigated (Fig. 5A,B). In consistent, indomethacin‐induced *Chop* mRNA upregulation was reduced by SAC pretreatment (Fig. 5C). Immunocytochemistry revealed that indomethacin changed cell morphology and increased the nuclear level of CHOP, both of which was partially reversed by SAC pretreatment (Fig. 5D,E). These data suggest that SAC protects hepatocyte apoptosis through reducing ER stress. Figure 6 is a schema illustrating how SAC protects indomethacin‐induced apoptosis of hepatocytes.

## Discussion

Nonsteroidal anti‐inflammatory drugs are among the most widely used drugs due to not only their pain and fever‐relieving effects, but also their availability either by prescription or over‐the‐counter [33]. NSAIDS‐associated hepatotoxicity, as reported in the literature, accounts for ~ 10% of all DILI [8, 34]. Moreover, several NSAIDS (benoxaprofen, ibufenac, and bromfenac) were withdrawn from the market due to their liver toxicity [35, 36]. At present, *N*‐acetylcysteine has been proved to be an antidote for DILI induced by acetaminophen as well as other drugs [37, 38, 39], and corticosteroids have been used to treat immune‐mediated drug reactions in DILI [40]. Nevertheless, the remedy for NSAIDS‐associated DILI is still lacking, and new drugs are desperately needed. The present study demonstrates that SAC alleviates indomethacin‐induced hepatocyte apoptosis, which sheds light on new treatments for DILI.

Studies on pharmacokinetics of SAC revealed high oral absorption in experimental animals, with the bioavailability value of 92.1–98% in rats, and 92% in dogs [41, 42]. Also, high bioavailability of SAC after oral intake was reported in healthy human volunteers [43]. Meanwhile, the clearance of SAC in humans is relatively slow. The plasma half‐life of SAC is > 10 h and the estimated clearance time is over 30 h [43], suggesting a potential long dosage interval. Toxicity tests revealed that the median lethal dose (LD50) of SAC upon oral administration in mice and rats was over 8.8 g·kg^−1^, and the LD50 on intraperitoneal injection in rats was comparable to that of essential amino acids [43]. The high bioavailability and low‐toxicity properties of SAC provide great virtues for its employment in animal studies as well as in future clinical trials.

The antioxidant feature of SAC has been intensively investigated, and much work has shown that SAC exerts its hepatoprotective function through mitigating oxidative stress. In carbon tetrachloride treated rats, SAC dose‐dependently inhibited lipid peroxidation and decreased serum levels of ALT and lactate dehydrogenase [44]. In Wistar rats insulted with hexavalent chromium, SAC protected against liver cell apoptosis by upregulating the hepatic expression of NF‐E2‐related factor 2, a crucial regulator of cellular antioxidant response [45]. However, in spite of these studies, little is known concerning the effects of SAC on ER stress in hepatocytes. The current study demonstrates that SAC abates indomethacin‐induced eIF2α phosphorylation, inhibits CHOP upregulation and its nuclear translocation, abrogates the activation of subsequent caspase 3 apoptotic pathway, and, finally, protects hepatocytes from apoptosis. To the best of our knowledge, this is the first study demonstrating the impact of SAC on ER stress in hepatocytes. Our data suggest that ER stress may be a latent therapeutic target for the treatment of DILI.

Another interesting finding is that inhibition of ER stress by 4‐PBA partially, but not fully, reversed indomethacin‐induced hepatocyte apoptosis, indicating that ER stress is among the various mechanisms by which indomethacin induces apoptosis of hepatocytes. Previously, indomethacin has been shown to enhance ROS generation and reduce intracellular antioxidant capacity in hepatocytes [46], evidence of the involvement of oxidative stress in indomethacin‐induced apoptosis. Given the potent antioxidative efficacy of SAC, it is presumable that SAC protects indomethacin‐insulted hepatocytes through reducing not only ER stress, but also oxidative stress.

The most conserved ER sensor inositol‐requiring enzyme 1α (IRE1α) is a transmembrane ER kinase as well as an endoribonuclease. Upon ER stress, activated IRE1α splices a small intron (26 bp) from the *Xbp1* mRNA, generating an active spliced XBP1 protein (XBP1s) [47]. XBP1s then translocates to cell nucleus and drives transcription of genes responsible for restoring protein homeostasis [14]. In the past two decades, XBP1 has been shown to be a survival factor in different categories of cells [48, 49, 50]. Moreover, our previous study found that overexpression of XBP1s by adenovirus abated hydroquinone‐induced CHOP upregulation, while knockdown of XBP1 resulted in significant increase of *Chop* mRNA level in the retinal pigment epithelium, suggesting a potential regulation of XBP1s on CHOP expression [51]. The present study demonstrates the upregulation of XBP1s protein as well as the splicing of *Xbp1* mRNA upon indomethacin treatment, evidence of IRE1α activation, and ER stress implication in indomethacin‐insulted hepatocytes. Nevertheless, the role of XBP1s on indomethacin‐induced apoptosis and the influence of SAC on XBP1 expression in hepatocytes are to be explored in our future experiments.

Taken together, the current study demonstrates that SAC alleviates ER stress and inhibits CHOP expression, thus protects hepatocyte apoptosis induced by indomethacin. SAC may present a potential new therapeutic agent for the treatment of DILI.

## Conflict of interest

The authors declare no conflict of interest.

## Author contributions

YR and PC conceived and designed the experiments; PC, MH, RC, FL, and HY acquired the data; PC, CC, MH, and YR analyzed and interpreted the data; PC, CC, and YR wrote the manuscript.

## Data Availability

The raw data of this study are available from the corresponding author on reasonable request.
